# The Relationship Between Fear of COVID-19 and Online Aggressive Behavior: A Moderated Mediation Model

**DOI:** 10.3389/fpsyg.2021.589615

**Published:** 2021-02-15

**Authors:** Baojuan Ye, Yadi Zeng, Hohjin Im, Mingfan Liu, Xinqiang Wang, Qiang Yang

**Affiliations:** ^1^Center of Mental Health Education and Research, School of Psychology, Jiangxi Normal University, Nanchang, China; ^2^Department of Psychological Science, University of California, Irvine, Irvine, CA, United States; ^3^School of Education, Jiangxi Normal University, Nanchang, China

**Keywords:** fear of COVID-19, moral disengagement, family cohesion, online aggressive behavior, Chinese college students, COVID-19, coronavirus

## Abstract

Amid the COVID-19 pandemic, fear has run rampant across the globe. To curb the spread of the virus, several governments have taken measures to drastically transition businesses, work, and schooling to virtual settings. While such transitions are warranted and well-intended, these measures may come with unforeseen consequences. Namely, one’s fear of COVID-19 may more readily manifest as aggressive behaviors in an otherwise incognito virtual social ecology. In the current research, a moderated mediation model examined the mechanisms underlying the relation between fear of COVID-19 and overt and relational aggressive online behavior among Chinese college students. Utilizing a large sample of Chinese college students (*N* = 2,799), results indicated that moral disengagement mediated the effect of fear of COVID-19 on college students’ overt and relational online aggressive behavior. A positive family cohesion buffered the effect of moral disengagement on relational aggressive behavior, but only for females. The findings, theoretical contributions, and practical implications of the present paper are also discussed.

## Introduction

In order to minimize the spread of COVID-19, the Chinese government advised citizens to stay home and avoid non-essential travel early in the pandemic. Under state-mandated orders for sheltering in place, much of social interactions and dissemination of news and information transitioned to online networks. Compared to the end of 2019, China’s Internet traffic had increased by approximately 50% by mid-2020 (Liu, 2020). Although the internet brings convenience to our lives, it is inevitably accompanied by deviant behavior (e.g., online aggressive behavior). For instance, recent evidence suggests that approximately 59.47% of Chinese college students have participated in online aggressive behavior at one time or another (Jin, 2018) across various platforms, such as via social media and gaming (Wright, 2020). Aggression commonly manifests in two forms, (1) overt (i.e., confrontational acts) and (2) relational aggression (i.e., social gossip and interpersonal damage) (Crick, 1996; Zhao and Gao, 2012). Both types of aggression can result in severe psychological (Guo, 2016; Pabian and Vandebosch, 2016) and physiological consequences to victims (Vaillancourt et al., 2017; Tozun and Babaoglu, 2018). Thus, while internet access has certainly made the transition to an isolated world amid the pandemic smoother, it has heightened our need to monitor the negative consequences of online aggression, particularly among the more tech-savvy youth.

Confronted by COVID-19’s veil of novelty and uncertainty shrouding the outcomes of the future, fear has been a natural response by many affected individuals (Wang J. X. et al., 2020). Although negative emotion has been documented to be related to engagement in aggressive behavior (e.g., Song, 2019), no study, to the best of our knowledge, has examined the relation between fear of COVID-19 and engagement in online aggressive behavior among Chinese college students. Therefore, the aims of the present study were to examine and test whether fear of COVID-19 was significantly related to online aggressive behavior among Chinese college students and the underlying mediating and moderating mechanisms in this association.

### Fear of COVID-19 and Online Aggressive Behavior

Fear has universally been documented and regarded as a key basic negative emotion, elusive in its influence on human behavior (e.g., Watson et al., 1988). During widespread events of public health emergencies, symptoms of negative mood disorders (e.g., fear, anxiety, depression) are common (Dai, 2014). Accordingly, fear has been documented to be pervasive and persistent amid COVID-19 (Wang J. X. et al., 2020). This atmosphere of fear, in turn, presents a troublesome preamble to the reality of the consequences befalling on the citizens’ mental and physical health through pervasive state of heightened stress (Wang Y. et al., 2020). However, it is unclear how individuals cope with the sudden influx of negative cognition and emotion and what the social consequences are.

In recent years, several studies have linked fear and aggression in humans (Simunovic et al., 2013; Halevy, 2017; Mifune et al., 2017). However, such findings have often been relegated to examining preemptive aggression in response to anticipated prosecution from an antagonizing party. Fear of COVID-19 presents a unique and qualitatively different scenario in which fear may manifest into aggression. People naturally gravitate, whether willingly or otherwise, to seek to manage emotional problems and mitigate negative outcomes (Zillmann et al., 1974; Larsen, 2000). As young adults, college students may often lack the necessary life experiences and skills to cope with novel problems and instead engage in maladaptive coping strategies, such as aggression, to mitigate their negative emotions (Bitler et al., 1994; Sprott and Doob, 2000; Song, 2019). Indeed, although aggression has negative social consequences (Sun et al., 2016), it is a common maladaptive coping mechanism (Bushman et al., 2001; Roberton et al., 2012) particularly when one loses agentic control over their environment and seeks out compensatory control (Marcus-Newhall et al., 2000; Shoss et al., 2015). Marcus-Newhall et al. (2000) proposed that in the absence of the ability to directly tackle the antagonizing force, individuals may be motivated to displace their negative emotions toward otherwise innocent others. The consequences of pervasive COVID-19 related negative affect may further be accentuated by the only available outlet to individuals under lockdown–the internet.

During the COVID-19 outbreak, the Chinese government mandated residents to shelter at home to curtail the spread of the virus. This inadvertently increased internet traffic as residents spent more time online in the absence of in-person interactions. The anonymity of the internet can serve as a shield to protect aggressors from immediate consequences (Moore et al., 2012), further waning the psychological restraint one may self-impose for normative social interactions. Indeed, individuals have been documented to resort to use of foul language to vent their negative emotions on anonymous platforms (Dang, 2017). The anonymity afforded to people surfing the web may inadvertently make it easier for students to engage in online aggressive behavior without the normal accompanying guilt and restraint, regardless of whether that aggression is overt or more indirect (i.e., relational). Individuals may also be more easily triggered by potential aggressors given that a negative emotional state promotes more biased and cynical evaluation of others (Beukeboom and Semin, 2006). Although not yet empirically tested, there is reasonable conceptual rationale to expect that COVID-19 induced fears may lead to greater online aggressive behavior. Because the COVID-19 pandemic leaves little room for individual agentic control, induces fear amongst the populace, and has also forced many residents to shelter at home with only the internet as the gateway toward social contact, the current pandemic may have concocted the necessary reagents to stir a rather contentious virtual social ecology for human interactions.

### Moral Disengagement as a Mediator

The relation between fear of COVID-19 and online aggressive behavior is unlikely to be a simple, direct one. The general aggression model (GAM) posits that personal and situational factors will influence internal states (Anderson and Bushman, 2002; Watts et al., 2017; Fang et al., 2020). Thus, drawing from GAM, moral disengagement may mediate the effect of fear of COVID-19 on online aggressive behavior. That is, as a moral guilt and regulation inhibitor, moral disengagement allows individuals to justify and reappraise their immoral actions, minimizing one’s perceived role in the outcome of their actions or at least reducing the apparent distress stemming from what they cause to others (Bandura et al., 1996; Bjärehed et al., 2020).

Prior studies have long documented that negative emotions (e.g., anger) are related to greater tendency to morally disengage (Jin et al., 2017), possibly as a self-protective measure against any consequences stemming from negative emotion-driven actions (Anderson and Bushman, 2002). As a stateful cognitive orientation, moral disengagement is mutable in response to emotions and internal factors (Moore, 2008). Further, prior studies have documented that individuals with higher levels of moral disengagement are more likely to aggress against others (Bussey et al., 2015; Zheng et al., 2016; Mazzone et al., 2019) in forms of overt and relational aggression (Zhao and Gao, 2012). With internet communication abundant among youths, online aggression is considered a natural derivative of traditional aggression (Wong-Lo et al., 2011). Indeed, moral disengagement and negative emotions have been linked to engagement in aggression in online settings through contentious online comments and cyberbullying (Pornari and Wood, 2010; Renati et al., 2012; Bussey et al., 2015; Runions and Bak, 2015; Wang et al., 2016; D’Errico and Paciello, 2018). Due to reduction of self-punishment and guilt, it may be easier for individuals to vent their emotions and stress on innocent people through negative online interactions (Pornari and Wood, 2010). Therefore, greater moral disengagement will likely lead to greater aggression and mediate the effect of fear of COVID-19.

### The Moderating Role of Family Cohesion

Due to government mandated home quarantines, college students in China found themselves situated at home with their families during the pandemic. The family dynamic plays an important role for individuals’ healthy psychological (Zhan and Li, 2019; Liu G. Z. et al., 2020; Liu T. et al., 2020), emotional (Olson et al., 1979, 1982), and individual development (Miller et al., 2000) through primary goal setting for successful achievement of a variety of basic, developmental, and crisis tasks (Skinner et al., 2000). Indeed, family cohesion comprises the emotional bonding between family members (Reeb et al., 2015) and serves as an important facet of proper socialization (Olson et al., 1979, 1982) which may be crucial in managing adaptive behavioral conduct and management. For instance, individuals who report experiencing higher levels of family cohesion and adaptability have also reported engaging in fewer problematic (Jiang et al., 2018) and aggressive behavior (Lu et al., 2019).

From the perspective of the organism-environment interaction model (e.g., Lerner et al., 2006), behavioral tendencies are formed and developed in the process of the interaction between individual and environmental factors. In our study, moral disengagement poses a risk factor for online aggressive behavior while family cohesion serves as a protective factor against risk (Bao et al., 2014). In other words, the effect of moral disengagement on both overt and relational aggression should be the highest for college students who report lower family cohesion within their households and lowest for those with high family cohesion. To date, no research, to the best of our knowledge, has examined family cohesion as a moderator of the indirect relationships between moral disengagement and online aggressive behavior.

### The Present Study

**FIGURE 1:**
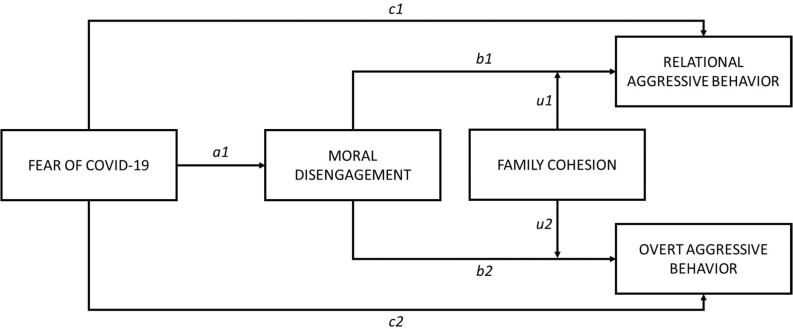
Conceptual moderated mediation model.

The current research tested the relation between fear of COVID-19 and online aggressive behavior and whether moral disengagement mediated this effect. Although several studies have been conducted examining the antecedents of aggression in online interactions, these prior studies have typically operationalized aggression as a unidimensional construct despite evidence of multidimensionality in how aggression manifests (Crick, 1996; Zhao and Gao, 2012). Zhao and Gao (2012) posit that aggression may be overt (i.e., *direct aggression*) or relational (i.e., *indirect aggression*), in which the former pertains to confrontational acts whereas the latter more encompasses social gossip and interpersonal damage. Although there is little reason to hypothesize different direction or size of effects of the aforementioned study variables on overt and relational aggression, we provide exploratory analyses separating male and female participants given the history of studies on how aggression differentially manifest across genders (e.g., Björkqvist, 2018). We also tested the buffering effect of family cohesion on the relation between moral disengagement and both overt and relational aggression (Figure 1). Based on the literature review, we proposed the following hypotheses:

*Hypothesis 1.* Fear of COVID-19 is positively related to (a) overt online aggression, (b) relational online aggression, and (c) moral disengagement.

*Hypothesis 2.* Moral disengagement is positively related to both (a) overt and (b) relational online aggression and mediates the effect of fear of COVID-19 on (c) overt and (d) relational online aggression.

*Hypothesis 3.* Family cohesion buffers the effect of moral disengagement on (a) overt aggression and (b) relational aggression.

## Materials and Methods

### Participants

Our study was approved by the Research Ethics Committee of the first author’s institution. All participating college students provided informed consent. A total of 2,799 Chinese college students (*M*_*age*_ = 19.63, *SD*_*age*_ = 1.23, range_*age*_ = 18–25, 70% female) anonymously completed the survey. Among the total sample, 1,402 (50.09%) were first years, 1,176 (42.02%) were second years, 128 (4.57%) were third years, and 93 (3.32%) were fourth years. A slight majority of the sample (*n* = 1,492, 53.5%) reported residency in urban settings.

### Measures

#### Fear of COVID-19

Fear of COVID-19 was measured via a self-report scale. Participants rated 9 items (e.g., “I worry about being infected by others”) on a five-point scale (1 = *never*, 5 = *always*), α = 0.91. Higher scores indicate higher level of fear. Confirmatory factor analysis (CFA) suggested that the one-factor model fit the data well; TLI = 0.99, CFI = 0.99, RMSEA = 0.05, 90% CI = [0.040, 0.054], SRMR = 0.02.

#### Moral Disengagement

Moral disengagement was measured via the Moral Disengagement Scale (MDS) [Detert et al., 2008; Chinese version revised by Wang and Yang (2010)], α = 0.90. Participants completed 26 items (e.g., “It is okay to tell small lies because they don’t really do any harm”) on a five-point scale (1 = *strongly disagree*, 5 = *strongly agree*) assessing eight dimensions of moral disengagement including (1) moral justification (4 items, e.g., “It is alright to fight to protect your friends”), (2) euphemistic labeling (3 items, e.g., “Talking about people behind their backs is just part of the game”), (3) advantageous comparison (3 items, e.g., “Stealing some money is not too serious compared to those who steal a lot of money”), (4) displacement of responsibility (3 items, e.g., “If someone is pressured into doing something, they shouldn’t be blamed for it”), (5) diffusion of responsibility (4 items, e.g., “You can’t blame a person who plays only a small part in the harm caused by a group”), (6) distortion of consequences (3 items, e.g., “Insults don’t really hurt anyone”), (7) dehumanization (3 items, e.g., “It is ok to treat badly someone who behaved like a ‘worm”’), and lastly (8) attribution of blame (3 items, e.g., “People who are mistreated have usually done things to deserve it”). Higher scores indicated greater moral disengagement. The Chinese version of the scale has previously been used with Chinese participants with good reliability and validity (Liu et al., 2014; Liu and Liu, 2020).

#### Family Cohesion

Family cohesion was measured via the cohesion subscale of the Family Adaptability and Cohesion Evaluation Scale [FACES; Olson et al., 1982; Chinese version revised by Fei et al. (1991)], α = 0.89. The scale consisted of 16 items (e.g., “When there are difficulties, family members will try their best to support each other”). Each item was rated on a 5-point scale (1 = *never*, 5 = *always*), with higher total scores indicating higher levels of family cohesion. The Family Cohesion Scale has been used with Chinese participants with good reliability and validity (Lin et al., 2018; Li et al., 2019; Li L. et al., 2020; Ye et al., 2019).

#### Online Aggressive Behavior

Online aggressive behavior was assessed by Adolescent Online Aggressive Behavior Scale (Zhao and Gao, 2012), α = 0.76. The scale assessed two dimensions of online aggressive behavior including overt aggression (7 items, e.g., “I often abuse others when I play online games”) and relational aggression (8 items, e.g., “I badmouth someone on the Internet with my friends”). All items were rated on a four-point Likert scale (1 = *never*, 4 = *always*). Higher scores indicated greater engagement in online aggressive behavior. The scale has been used with Chinese participants with good reliability and validity (Zheng et al., 2016; Jin, 2018).

### Procedure

Due to government issued orders to shelter-at-home during China’s early stages of the COVID-19 pandemic, questionnaires were distributed electronically via WeChat and QQ. The survey was hosted on Survey Star (Changsha Ranxing Science and Technology, Shanghai, China) from February 16 to March 1, 2020 and all responses were anonymous. Participation in the study was entirely voluntary and no compensation was given for their participation.

## Results

### Preliminary Analyses

Fear of COVID-19 was positively correlated with both moral disengagement and online aggressive behavior while negatively correlated with family cohesion (Table 1). Moral disengagement was positively correlated with online aggressive behavior and negatively correlated with family cohesion. Family cohesion was negatively correlated with online aggressive behavior.

**TABLE 1:** Bivariate correlations of the study variables.

	*M ± SD*	1	2	3	4	5
1. Age	19.64 ± 1.24	–				
2. Fear of COVID	1.88 ± 0.66	0.18***	–			
3. Moral disengagement	1.37 ± 0.41	0.09***	0.23***	–		
4. Online aggressive behavior	1.05 ± 0.10	0.09***	0.16***	0.41***	–	
5. Family cohesion	4.14 ± 0.66	−0.04*	−0.08***	−0.13***	−0.14***	–

N = 2,799, *p < 0.05, ***p < 0.001.

### Testing for Mediation Effect and Moderated Mediation Effect

The conceptual model (Figure 1) was examined in Mplus 7.4 (Muthén and Muthén, 2015) and path coefficients are given in Table 2. Due to evident differences in the study variables across demographic factors (see Table 1 and Appendix Tables A,B), each path controlled for gender, age, and urban/rural setting. The examined model using the total sample indicated good fit for overt aggression (RMSEA = 0.041, 90% CI [0.038, 0.044], CFI = 0.978, TLI = 0.973, SRMR = 0.039) and relational aggression (RMSEA = 0.038, 90% CI [0.035, 0.041], CFI = 0.981, TLI = 0.976, SRMR = 0.035) based on field-normative thresholds (Kline, 2011; Hoyle, 2012) (for visual representation, see Appendix Figures A,B). Fear of COVID-19 was positively related to moral disengagement but only directly related to relational aggression, supporting Hypotheses 1b-c while rejecting 1a. Moral disengagement was positively related to both overt and relational aggression (*β* from 0.327 to 0.461, *p* both < 0.001), supporting Hypotheses 2a-b. Moral disengagement also, respectively, fully and partially mediated the effects of fear of COVID-19 on overt and relational aggression (*β* from 0.083 to 0.118, *p* < 0.001), supporting Hypotheses 2c-d. However, family cohesion only moderated the effect of moral disengagement on relational aggression (*β* = −0.056, *p* = 0.019) and not overt aggression (*β* = −0.042, *p* = 0.065), supporting Hypothesis 3b and rejecting Hypothesis 3a.

**TABLE 2:** Path coefficients across the total sample, male sample, and female sample.

	a		b		c		u	Indirect Effect
							
Predictor	*β*	*M*	*β*	Y	*β*	U	*β*	*β*
***Total Sample***								
Fear COVID	0.254***	Moral Dis.	0.327***	Overt	0.039	Fam. Coh.	−0.042	0.083***
Fear COVID	0.255***	Moral Dis.	0.461***	Relat.	0.077**	Fam. Coh.	−0.056*	0.118***
***Male***								
Fear COVID	0.255***	Moral Dis.	0.345***	Overt	0.073	Fam. Coh.	−0.031	0.088***
Fear COVID	0.255***	Moral Dis.	0.361***	Relat.	0.096*	Fam. Coh.	−0.013	0.092***
***Female***								
Fear COVID	0.268***	Moral Dis.	0.692***	Overt	−0.014	Fam. Coh.	−0.064	0.185***
Fear COVID	0.269***	Moral Dis.	0.740***	Relat.	0.064	Fam. Coh.	−0.162***	0.199***

Fear COVID = Fear of COVID-19; Moral Dis. = Moral Disengagement; Overt = Overt Aggression; Relat. = Relational Aggression; Fam.Coh. = Family Cohesion; *p < 0.05, **p < 0.01, ***p < 0.001.

The male sample model indicated good fit for both overt (RMSEA = 0.039, 90% CI [0.032, 0.045], CFI = 0.986, TLI = 0.982, SRMR = 0.046) and relational aggression (RMSEA = 0.037, 90% CI [0.030, 0.043], CFI = 0.987, TLI = 0.984, SRMR = 0.043) (for visual representation, see Appendix Figures C,D). Across both models, moral disengagement mediated the effect of fear of COVID-19 on aggression (*β* from 0.088 to 0.092, *p* < 0.001). Fear of COVID-19 only showed a small direct effect for relational aggression (*β* = 0.096, *p* = 0.013) but not overt aggression (*β* = 0.073, *p* = 0.062). Family cohesion, however, did not moderate the path from moral disengagement to aggression (*β* from −0.013 to −0.031, *p* from 0.418 to 0.737). The female sample model likewise indicated good fit for both overt (RMSEA = 0.037, 90% CI [0.033, 0.041], CFI = 0.980, TLI = 0.975, SRMR = 0.037) and relational aggression (RMSEA = 0.036, 90% CI [0.033, 0.040], CFI = 0.981, TLI = 0.976, SRMR = 0.038) (for visual representation, see Appendix Figures C, D). Compared to the male sample models, moral disengagement fully mediated the effect of fear of COVID-19 on aggression (*β* from 0.185 to 0.199, *p* < 0.001). Family cohesion buffered the effect of moral disengagement on aggression, but only for relational aggression (*β* = −0.162, *p* < 0.001) and not overt aggression (*β* = −0.064, *p* = 0.276), partially supporting Hypothesis 3b.

## Discussion

Findings from this study showed that fear of COVID-19 was positively related to online aggressive behavior via moral disengagement. This finding, however, showed large variance across males and females. These results help to highlight the psychological processes of how fear of COVID-19 may lead to more online aggressive behavior among college students and has key implications for decreasing college students’ online aggressive behavior amid the ongoing pandemic.

### The Relationship Between Fear of COVID-19 and Online Aggressive Behavior

Results partially supported the hypothesis that fear of COVID-19 would be a positive correlate of online aggressive behavior, consistent with general findings on negative affect and aggression (Jiang, 2012; Song, 2019). Specifically, the direct effect of fear of COVID-19 on online aggressive behavior was only significant for males, and only for relational aggressive behavior. We originally hypothesized that the accumulation of fear of COVID-19 would lead individuals to aggress others in virtual spaces to relieve their negative affect and possibly cope with COVID-19 related concerns and stressors (Ma and Lei, 2010). The inconsistent effects observed in this study indicate that COVID-19 related fears may be small factors on aggression but promotes the activation of moral disengagement – a key antecedent of aggression. This no doubt raises the need to monitor aggression in virtual spaces, as the spread of the virus has forced much of the population to transition to online for education or work. Indeed, the anonymity afforded to those in virtual spaces (Moore et al., 2012) can induce greater moral disengagement that promote different behavioral and emotional expressions on the internet than what one would otherwise partake in reality (Li, 2011; Li et al., 2012).

### The Mediating Role of Moral Disengagement

The strong mediating effect of moral disengagement was robust across genders and subcategories of aggression behavior. The positive effect of fear on moral disengagement was consistent with prior literature (e.g., Caprara et al., 2013; Chen, 2016) and may reflect the cognitive motivation to seek methods to protect oneself when threatened (e.g., Cosmides and Tooby, 2000). Amid the pandemic, negative emotions from COVID-19 related concerns are likely to run high and may activate moral disengagement as a cognitive defensive mechanism (Paciello et al., 2012; Fida et al., 2015). The positive effect of moral disengagement on aggressive behavior was also consistent with prior studies on aggression (Runions and Bak, 2015; Liu and Liu, 2020) and cyber-bullying (Wang et al., 2016). In this study, moral disengagement likely served to justify or neutralize the self-restraining values that otherwise would deem antagonistic behavior as morally reprehensible. As online interactions commonly filter immediate feedback (e.g., facial, verbal) that normally trigger processing of guilt (Josnson, 2003), these online spaces may further provide fertile ground for easy moral disengagement.

It is worth noting, however, the prevalent variation in findings within our sample. Notably, moral disengagement fully mediated the relation between fear of COVID-19 and aggressive behavior for females. One possible explanation is the myriad of cultural norms that befall young girls in China. For instance, in China, it is generally less socially and culturally acceptable for females to exhibit aggressive behaviors (Li X. et al., 2020). Bound by the responsibility to adhere to social norms, it is plausible that females may have felt compelled to morally disengage and cognitively justify their aggressive behaviors more so than their male counterparts. This may partly also explain the larger effect moral disengagement had on aggressive behavior among female participants compared to male participants. That is, for females, moral disengagement may serve as a stronger requisite for surmounting the social restraints of “proper conduct” to aggress others compared to males who face less social and cultural demands. Another explanation may be that females may have been more empathetic of the individual they were interacting with, even with the anonymity that accompanies online social media. Indeed, as cyberbullies report lower levels of empathy (Renati et al., 2012), whether there are gender differences in how empathy may necessitate greater moral disengagement may be examined in the future.

The necessity for moral disengagement to overcome strict social guidelines may also partly explain the full mediation effect for overt aggression among males. Despite aggression being relatively more permissible for males than females in China (Li X. et al., 2020), because overt aggression entails strong, direct antagonization (Zhao and Gao, 2012), it may be such that moral disengagement still remains a strict requisite for more direct aggression among males. Nonetheless, the effect of moral disengagement on online aggression requires further cross-cultural replication. Specifically, the effect of moral disengagement on online aggressive behavior has been evidenced to be larger in Chinese culture compared to Western cultures (Wang et al., 2014). Future studies may test the robustness of the examined model across different cultures and societies.

### The Moderating Role of Family Cohesion

The current study further found a partial support for the buffering effect of family cohesion on online aggressive behavior in accordance with the risk buffering model (Lerner et al., 2006; Bao et al., 2014). The conjecture was that those with greater family cohesion would be able to utilize their family as a resource for stress management in contrast to those with lower family cohesion who may feel the need to resort to online aggression as a form of maladaptive coping. Indeed, prior studies have evidenced that college students with weaker familial cohesion have reported feeling lonelier (Ren, 2020) and are more likely to attack others online (Sun et al., 2017). However, our study found that family cohesion provided limited protection. Specifically, this buffering effect was only significant among females against relational aggressive behavior. Like the case with moral disengagement, the Chinese social norms for gendered conduct may provide some insight. In China, males are often preached values of strength and traditional masculinity (e.g., “a man should strengthen himself [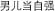],” “real men do not easily cry [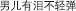]”) and given lower emotional attention from parents during childhood development (Ye et al., 2020). Thus, one possibility may be that males may find it more difficult to utilize their families as resources for stress management compared to females who may readily receive more emotional support (Ye et al., 2020) and cope through productive dialogue (Tamres et al., 2002). This echoes the ongoing issue of toxic masculinity in negative mental health outcomes (Parent et al., 2019). Although the topic of toxic masculinity in China has been given less attention than in their Western neighbors, recent evidence has alluded to the prevalence of gender differences in how males and females are given familial attention and support (e.g., Ye et al., 2020). Should males be more hesitant in seeking adaptive coping strategies, it may be worth examining alternative modes with which they can obtain proper resources anonymously online to not incur any cultural or social backlash. Future research can further this inquiry by examining how cultural and social expectations may inhibit individuals from seeking or utilizing available resources in stress management.

### Limitations and Future Directions

Several limitations must be considered when interpreting the results of the present study. Firstly, the present study was cross-sectional and causal inference is limited. However, given that experimentally manipulating fear of COVID-19 may result in artificial fearmongering or downplaying of COVID-19, we recommend caution in designing future experimental studies. Future studies may instead seek to design longitudinal studies to better infer the temporal relation of the paths in this model. Secondly, all variables included in this study were measured via self-report scales. For topics like cyberbullying, participants may be motivated to underreport their actual engagement. Future studies may try to collect data from multiple informants (e.g., family members) or opt to using text-analysis methods to further nuance the current findings. It may also be interesting to examine whether there are parental support differences (i.e., support from mother vs. support from father) in mitigating aggression. Thirdly, the sample used in this study were entirely Chinese college students, limiting the generalizability of findings across cultures. Fourthly, individual factors (e.g., personality) were not measured in this study. In light of prior studies documenting personality to be related to aggression (García-Sancho et al., 2017; Bresin, 2019), future studies may aim to expand the current model by incorporating such variables.

Despite these limitations, the current study contributes to the body of literature by providing a conceptual basis for designing social interventions. In particular, academics and policymakers may seek to design interventions that address the negative emotions stemming from the ongoing pandemic as well as better engaging with one’s moral values in online social interactions. Additionally, future studies may examine to what extent related, but distinct negative emotions (e.g., anger) may also result in increased aggression.

## Conclusion

Although further replication and extension efforts are advised, this study represents an important step forward in unpacking how fear of COVID-19 may be related to the manifestation of online aggressive behavior among Chinese college students via moral disengagement. Although many societies have been working toward reopening their businesses and schools, the angst of COVID-19 will likely linger for much longer. For populations that regularly engage in social interactions in the virtual space (e.g., children, adolescents), self-monitoring may be crucial for maintaining a civil virtual social ecology. Moreover, the limited buffering effect of family cohesion for females in relational aggression warrants further examination of how males and females may respond to different types of stress coping resources. As our social lives will inevitable become more intertwined with the digital world, future research may help us to better understand how we may mitigate the manifestation of negative behaviors online.

## Data Availability Statement

The raw data supporting the conclusions of this article will be made available by the authors, without undue reservation.

## Ethics Statement

The studies involving human participants were reviewed and approved by the Research Ethics Committee of Jiangxi Normal University. Participants all provided informed consent prior to participating in the current study.

## Author Contributions

BY acted as the Principal Investigator and oversaw the study in its inception to completion. BY, YZ, ML, XW, and QY were responsible for data collection, writing the manuscript, and conceptualizing the models. HI was responsible for writing the manuscript and conceptualizing the models. All authors contributed to the article and approved the submitted version.

## Conflict of Interest

The authors declare that the research was conducted in the absence of any commercial or financial relationships that could be construed as a potential conflict of interest.

## Figures and Tables

**FIGURE A:**
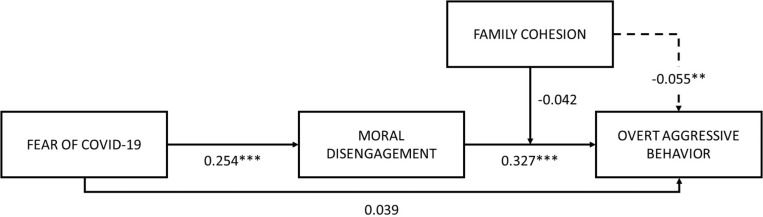
Model 1 Total sample on overt aggression behavior. RMSEA = 0.041, 90% CI [0.038, 0.044], CFI = 0.978, TLI = 0.973, SRMR = 0.039.

**FIGURE B:**
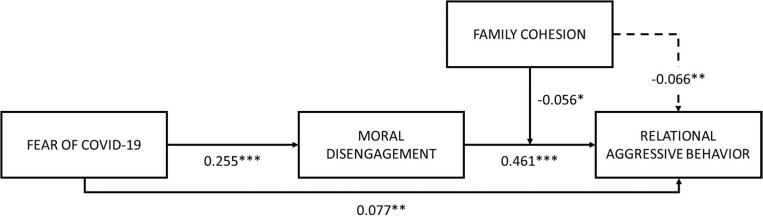
Model 2 Total sample on relational aggression behavior. *RMSEA* = *0.038, 90% CI [0.035, 0.041], CFI* = *0.981, TLI* = *0.976, SRMR* = *0.035*.

**FIGURE C:**
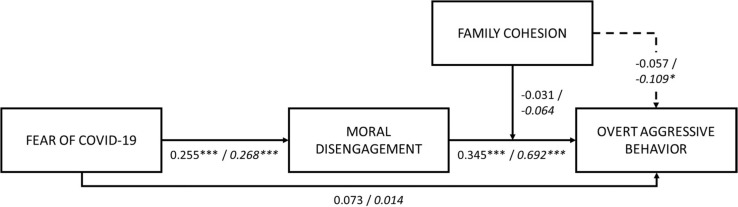
Model 3 Male/Female samples on overt aggression behavior. Male sample (RMSEA = 0.039, 90%CI [0.032, 0.045], CFI = 0.986, TLI = 0.982, SRMR = 0.046); Female sample (RMSEA = 0.037, 90%CI [0.033, 0.041], CFI = 0.980, TLI = 0.975, SRMR = 0.037); female sample coefficients given in *italics*.

**FIGURE D:**
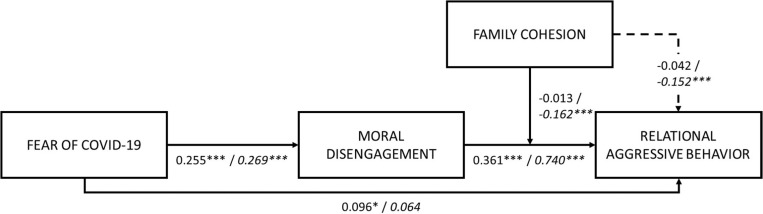
Model 4 Male/Female samples on relational aggression behavior. Male sample (RMSEA = 0.037, 90%CI [0.030, 0.043], CFI = 0.987, TLI = 0.984, SRMR = 0.043); Female sample (RMSEA = 0.036, 90%CI [0.033, 0.040], CFI = 0.981, TLI = 0.976, SRMR = 0.038); female sample coefficients given in *italics*.

## Appendix

**TABLE A:** Gender differences across study variables.

	Male		Female					
	*M*	*SD*	*M*	*SD*	*t*	df	*p*	*g*
Fear of COVID	1.81	0.75	1.91	0.62	−3.74	2797	<0.001	−0.151
Moral disengagement	1.47	0.55	1.34	0.33	7.79	2797	<0.001	0.319
Online aggressive behavior	1.08	0.17	1.04	0.05	10.05	2797	<0.001	0.395
Family cohesion	4.07	0.69	4.16	0.65	−3.51	2797	<0.001	−0.136

*Males n = 821; Females n = 1978; g = Hedge’s g.*

**TABLE B:** Environmental differences across study variables.

	Urban		Rural					
	*M*	*SD*	*M*	*SD*	*t*	df	*p*	*g*
Fear of COVID	1.84	0.65	1.92	0.67	−3.03	2797	0.003	−0.120
Moral disengagement	1.35	0.40	1.41	0.42	−4.04	2797	<0.001	−0.145
Online aggressive behavior	1.04	0.10	1.06	0.11	−3.89	2797	<0.001	−0.187
Family cohesion	4.17	0.68	4.09	0.63	3.27	2797	0.001	0.124

*Urban n = 1492; Rural n = 1307; g = Hedge’s g.*
